# Impact of the 2020 New Zealand COVID‐19 lockdown on participants in a community‐based, peer‐led fall prevention program

**DOI:** 10.1111/ajag.13040

**Published:** 2022-02-04

**Authors:** Jim Smith, Finn Whittington, Charlotte Ackermann, Regan Clarke, Georgia Hoten‐Walker, Yezen Kubba, Chin Low, Karina Partridge, Chris Wang, John D. Dockerty, Linda Robertson, Leigh Hale, Debra L. Waters

**Affiliations:** ^1^ Department of Preventive and Social Medicine Dunedin School of Medicine University of Otago Dunedin New Zealand; ^2^ Otago Polytechnic Dunedin New Zealand; ^3^ School of Physiotherapy University of Otago Dunedin New Zealand; ^4^ Department of Medicine Dunedin School of Medicine University of Otago Dunedin New Zealand

**Keywords:** accidental falls, aged, COVID‐19, physical fitness, social isolation

## Abstract

**Objectives:**

To explore the impacts of the 2020 New Zealand COVID‐19 lockdown on peer‐led Steady as You Go (SAYGO) fall prevention exercise classes and members, and to develop recommendations for mitigating impacts during future lockdowns.

**Methods:**

Semi‐structured phone interviews were conducted with 20 SAYGO program participants and managers following the first COVID‐19 lockdown in New Zealand. Interviews were audio‐recorded, transcribed verbatim and analysed using the General Inductive Approach.

**Results:**

Participants were between 67 and 88 years of age, predominantly female (90%) and NZ European (80%), with one participant identifying as NZ Māori. Three themes were constructed from the analysis: *Personal Function and Well*‐*Being*, *Class Functioning and Logistics*, and *Future Strategies for Classes During Prospective Lockdowns*. Participants used a range of strategies to stay connected with each other and continue the SAYGO exercises at home. Most participants and peer‐leaders reported that they maintained physical function during lockdown, although some had feelings of psychological distress and social isolation. Contact systems and resource distribution varied substantially between groups. Classes resumed post‐lockdown with only minor modifications and slightly decreased attendance.

**Conclusions:**

Overall, members of this peer‐led model of fall prevention classes demonstrated resilience during the COVID‐19 lockdown, despite some challenges. We propose three recommendations to address the challenges of maintaining existing peer‐led exercise classes in the context of prospective lockdowns: (1) develop a comprehensive contact detail register and plans for each group; (2) delivery of modified exercise classes remotely over lockdown; and (3) implementation of a nationwide IT education and resource program for older adults.

**Policy Impact**

A peer‐led, community‐based exercise model supported social connections and encouraged physical function in older adults during COVID‐19 lockdown in New Zealand. This model could be enhanced through classes creating comprehensive contact registers and a national roll‐out of ubiquitous technology and IT education designed for older people to facilitate social interactions remotely.

## 1 INTRODUCTION

Globally, consequences of the COVID‐19 pandemic and large‐scale lockdowns are continuing to emerge, with reports of disproportionately negative impacts on the morbidity and mortality of older adults.^1^, ^2^ Aotearoa New Zealand (NZ) implemented very strict restrictions, with only essential contact allowed during the height of the first nationwide lockdown (23/03/2020 to 13/05/2020). Further restrictions were implemented for vulnerable populations, including persons aged over 70 years, who were advised to stay at home before formal restrictions began.

International data suggest that older adults have suffered poorer quality of life and reduced physical activity as a result of COVID‐19, even in the absence of a strict nationwide lockdown.^3^, ^4^ Data from the United Kingdom (UK) found that only 25.4% of respondents reported a decrease in physical activity, albeit with less severe lockdowns compared to NZ.^5^ These data showed that decreased activity was associated with living alone and having a high self‐perceived health risk, although whether this decreased activity was associated with decreased function, increased falls, or mortality is unknown.^5^

Alongside physical health, the psychosocial impacts of large‐scale lockdowns are another concern. Social isolation is an important determinant of quality of life and overall health for older adults.^6^ Negative mental health effects appear to be a consequence of both the COVID‐19 pandemic itself, and associated lockdowns, with studies identifying increased rates of anxiety, depression and loneliness, and disrupted sleep during periods of lockdown.^7^, ^8^ Older adults may, however, be more resilient to the effects of social isolation than younger adults, due to positive self‐perceptions of ageing and self‐efficacy.^9^ With the obvious challenges to psychosocial health posed by COVID‐19 restrictions and social isolation, community connections are integral to maintaining well‐being during these stressful events.

The Steady As You Go (SAYGO) fall prevention program uses a unique model to improve long‐term adherence to weekly 1‐h strength and balance classes, being led by trained peer‐leaders in their communities. This model has shown efficacy in reducing falls, as well as promoting social cohesion between older adults.^10^, ^11^ The SAYGO classes have been widely implemented throughout NZ since 2003, now with 282 peer‐led groups and around 4500 members nationwide (C. Tarry, 2021, personal communication). A peer‐led model facilitates long‐term adherence through strong, non‐hierarchical social relationships, which promote cohesion and accountability in a member‐driven class environment. Peer‐led fall prevention classes in NZ enhance the physical, social and mental health of older adults; however, how these groups were impacted during the COVID lockdown is largely unknown.^10^, ^11^, ^12^, ^13^

Our study explored the experiences of SAYGO group members, peer‐leaders and managers as they adapted to the unique challenges posed by COVID‐19 lockdowns in NZ. We have used these findings to make recommendations for future lockdown situations both nationally and internationally.

## 2 METHODS

Semi‐structured interviews were performed to explore the experiences of individual SAYGO class members and peer‐leaders, and fall prevention managers from Age Concern, the co‐ordinating body of SAYGO classes in NZ. Data were analysed using the General Inductive Approach.^14^ We have adhered to the consolidated criteria for reporting qualitative research (COREQ) guidelines. This study was approved by the University of Otago Human Ethics Committee (Health H20/112), and we obtained consent verbally and by‐proxy signed consent forms.

### 2.1 Study population

Participants were recruited using snowball sampling via Age Concern co‐ordinators and SAYGO peer‐leaders. Eligible participants included SAYGO class members or peer‐leaders from the Otago/Southland and Wellington regions, aged 65 years or older. Due to the practicalities of conducting phone interviews, individuals with severe hearing impairment were excluded, as well as those with severe mobility impairments and those who could not consent for whatever reason. Ten peer‐leaders, eight class members and two Age Concern managers volunteered to participate in the study.

### 2.2 Research team reflexivity

The research team were 10 final‐year medical students and four established researchers. The medical student interviewers were trained and mentored by experienced qualitative team members (Authors LR, LH, DLW).

### 2.3 Data collection

Three interviewers (Authors GH‐W, YK,CL) performed all interviews between April and May 2021. Initially, participants were contacted by telephone to schedule an interview and to obtain verbal consent. A single, one‐on‐one interview was then conducted by a second Zoom telephone or video‐call (Zoom Video Communications, Inc.). The interviewers conducted semi‐structured interviews that explored the perceptions and experiences of participants regarding the impact of the COVID‐19 lockdown on classes and members; interactions between members and participants; and recommendations as to how classes should respond in the context of future lockdowns (Table S1). Demographic information included age, gender, ethnicity and a subjective rating of general health. Interviews were recorded using Zoom and transcribed using Otter.ai transcription software (Otter.ai, Inc.). The transcribed interviews were checked for accuracy by the research team against the audio‐recordings. Processed transcripts were not returned to participants, but were verified in a group feedback session at the end of the study.

### 2.4 Thematic analysis

As this was an evaluative study, data were analysed using an evaluative approach, the General Inductive Approach.^14^ In this pragmatic approach, raw data are systematically coded to establish a panel of core themes and subthemes that have a direct connection to the research objectives.^14^ Four research team members (Authors CA, RC,KP,CW) checked the transcribed interviews for accuracy against the audio‐recordings and undertook preliminary coding of the data. This was done by multiple reading of transcripts and identification of key concepts through several discussions. Independent parallel coding of each transcript was performed by two other research members (Authors JS, FW), blinded to the preliminary codes. Further discussion finalised the coding guide, which was then applied across all transcripts. Theme and subtheme generation was an iterative process, where themes were identified from coded transcripts via group consensus.

## 3 RESULTS

### 3.1 Participants

Initially, 24 participants volunteered, but only 20 were included in the final analysis (two were unable to be subsequently contacted, one declined to participate, and one was not involved with SAYGO during the COVID‐19 lockdown). Demographic details are listed in Table 1. Participants were predominantly female (*n* = 18, 90%) and of NZ European (*n* = 16, 80%) ethnicity, with only one participant identifying as NZ Māori. Ages ranged from 67 to 88 years. Participants were from three SAYGO groups: Dunedin (*n* = 5), Port Chalmers (*n* = 8) and Wellington (*n* = 7). The subjective general health of participants was rated as very good, with 80% reporting a general health rating of very good (*n* = 13) or excellent (*n* = 3); no participants considered themselves to be in poor health.

**TABLE 1:** Baseline characteristics for all study participants

Characteristic	Participants (*n*)	%
Age (years)		
65–69	2	10
70–74	9	45
75–79	3	15
≥80	6	30
Gender		
Male	2	10
Female	18	90
Ethnicity		
NZ European	16	80
NZ Māori	1	5
Other European	3	15
Location		
Dunedin	5	25
Port Chalmers	8	40
Wellington	7	35
Role		
Class member	8	40
Peer leader	10	50
Age Concern manager	2	10
Health rating		
Poor	0	0
Fair	2	10
Good	2	10
Very good	13	65
Excellent	3	15

Three themes were constructed from the data analysis: *Personal Function and Well*‐*Being*, *Class Functioning and Logistics*, and *Future Strategies for Classes During Prospective Lockdowns*, and these are described below along with their associated subthemes.

### 3.2 *Personal function and well*‐*being*

Alterations in individual function and well‐being over lockdown, both physical and psychological, emerged as a major theme amongst our cohort.

#### 3.2.1 Physical activity and conditioning

Strict lockdown restrictions prompted changes in activity for all participants, including the cessation of SAYGO and other regular exercise classes. Despite this, most (95%) participants reported being able to maintain, or even increase, their overall levels of activity during lockdown. Alternative forms of exercise, including walking, yoga, modified SAYGO exercises, and home‐based activity (e.g. stationary cycling, weight training) were deployed. Walking was the predominant form of regular exercise, with many reporting an increase in walking volume during lockdown. As a result, participants (90%) reported maintenance of physical functions, including improved physical fitness in some cases:

**PL:** ‘I felt quite fit, fitter after the lockdown really…. I actually got better afterwards because I probably did more exercise’.

All participants were able to resume SAYGO classes post‐lockdown, most without any obvious deconditioning or concerns reported. Two participants, however, said their physical function had decreased, which they attributed to progression of their underlying osteoarthritis.

#### 3.2.2 *Psychological and social well*‐*being*

Most participants reported coping relatively well over lockdown with regard to mental well‐being. There was, however, a generalised element of fear and anxiety with respect to COVID‐19 and the uncertainties of lockdown. Strong familial and peer‐support networks outside of SAYGO were highlighted by participants as protective factors for coping with the mental stressors of lockdown. One participant acknowledged that they coped very well due to their underlying propensity to lead an independent life, stating:

**CM:** ‘I'm perfectly happy to not have to have people around me…I'm not a person you could interview about the difficulties and I don't mind being alone in my house or not talking to people. I'm perfectly happy finding things to do or occupying my time in a number of ways and so it didn't trouble me’.

Several individuals described considerable mental distress and the negative impacts of social isolation on themselves or their peers. For example, one person said:

**PL:** ‘mentally it is quite distressing to be on your own’.

Those who reported mental distress tended to live alone and reported higher levels of perceived social isolation. For them, SAYGO classes were an important social support prior to lockdown and the loss of this support was concerning:

**PL:** ‘I think a lot of us were very anxious…we were all in the same boat. A lot of us on our own…living by ourselves. And a lot of us just need the company that this class [SAYGO] gives us as well as the exercises. The social side is equally important really’.

The impacts of losing contact with the SAYGO group extended to members who felt otherwise well‐supported during lockdown:

**PL:** ‘we hadn't realised how important that level of social contact was in our lives…we just found it was this huge gap from those kinds of weekly activities, where…you're not necessarily close friends with these people, but you meet them on a regular basis and just cutting out that level really made us both feel quite depressed’.

Following the resumption of SAYGO classes post‐lockdown, participants noted relief and improved well‐being with the return of regular in‐person social interaction.

### 3.3 Class functioning and logistics

Aspects of class functioning were impacted during and after lockdown, including contact between class members, performing informal SAYGO equivalent exercises and the introduction of additional precautions once classes resumed.

#### 3.3.1 Contact between SAYGO members

Contact between peer‐leaders and class members varied by group, ranging from no contact at all to weekly phone contact. Some peer‐leaders expressed that contacting participants was a challenge during lockdown due to a lack of contact information or limited resources, as many members did not have regular email access. For most SAYGO groups, contact with members was focused on logistical issues, but some expanded this to involve well‐being checks with members, which was often peer‐leader initiated and focused on providing social support:

**PL:** ‘Well, I suppose it sort of stemmed from my own feeling of being really cut off… How difficult it [COVID‐19 lockdown] was didn't really emerge straightaway, but I kind of guessed it wasn't going to be good…it was all such an uncertain time and I just couldn't bear the thought that this thing [SAYGO] that I find so beneficial and helpful, might kind of dwindle away; that sense of having those particular groups. So, I talked to the other two peer‐leaders in my class, and we kind of divided up the class amongst us and assigned each other people that we would keep in touch with…’

Several peer‐leaders felt class members did not require regular contact, as participants would often be in regular contact with pre‐existing friends or neighbours who were also involved in the SAYGO program. No concerns were raised during well‐being checks; however, many appreciated the social contact. Participants in regular contact reported that their relationships grew stronger over lockdown. Age Concern managers regularly contacted peer‐leaders to provide support and resources for distribution to members, such as shopping education and food vouchers, but resources were often difficult to pass on due to limited email use amongst class members. One peer‐leader physically mailed resources from Age Concern to class members to address this.

#### 3.3.2 SAYGO exercise equivalents

With no formal exercise classes over the most strict levels lockdown, many participants performed SAYGO exercise equivalents, either individually or in small groups. Some performed exercises from memory, whilst several members had access to the SAYGO class CD, which could be followed at home. Some used technology to follow the CD exercises with peers, such as FaceTime. One participant performed the exercises with their neighbour from opposite sides of their shared fence. One Age Concern manager uploaded a video of SAYGO exercises to YouTube, altered so they could be performed safely at home.

#### 3.3.3 Class functioning

The SAYGO classes resumed when lockdown was eased, contingent on group size and the ability to maintain social distancing in venues. Public health precautions were introduced to classes, including hand sanitiser use, social distancing, and contact tracing. Shared exercise equipment was not used due to contamination concerns. Despite these precautions, class structure remained the same with no necessity to change the level of difficulty post‐lockdown. As is typical for SAYGO classes, members were encouraged to exercise at their own pace and take breaks as required. In all groups, a gradual return of attendance was noted post‐lockdown, taking several weeks to return to near pre‐lockdown levels due to initial anxiety and uncertainty around personal safety.

**CM:** ‘Quite a few people came back…but it took a couple of weeks…everyone’s just a bit more careful…to ensure [it’s] safe’.

### 3.4 Future strategies for prospective lockdowns

Three major areas were identified by our respondents that need improvements for future lockdowns: intra‐group contact systems; remote access to SAYGO exercise equivalents; and access to technology, including information technology (IT) education.

#### 3.4.1 SAYGO group contact systems

Contact systems were highlighted as a major area of concern for several members of our cohort, particularly in groups reporting minimal or ineffective contact over lockdown:

**PL: ‘**People don't have computers or internet…some don't even have mobile phones…I felt almost a little bit helpless when it came to sharing the information during COVID’.

For one group, the lack of a formal phone list effectively prevented any contact between class members. A preference was expressed for more regular and structured interactions, with a greater focus on well‐being checks over discussions about logistics. Indeed, those members who received regular contact with a fellow SAYGO member that focussed on general well‐being reported a strengthening of those relationships over lockdown and greater overall levels of perceived social support.

#### 3.4.2 Remote accessibility to exercise alternatives

Increased remote accessibility to SAYGO exercises was also desired through initiatives such as the SAYGO CD, instructive YouTube videos or broadcast on public television. Participants expressed the view that these strategies could facilitate continued exercise over lockdown and prevent deconditioning, whilst retaining an important element of social accountability:

**CM: ‘**People should have the CD and a buddy system would be really good…If they can play the CD at home and link it on the phone with somebody, I'd say [they’re] far more likely to do it than on their own, because a lot of them don't have that motivation all the time, but with somebody else, it's very different’.

Having direct peer accountability was highlighted by some participants as an essential contributor to maintaining their physical activity whilst isolated during lockdown.

#### 3.4.3 Technology and IT education

Alongside the aforementioned suggestions, the value of IT education was noted for facilitating accessibility to interactive SAYGO equivalents from afar, as well as a range of useful online resources and the capacity to provide remote, face‐to‐face, social interaction:

**PL:** ‘To me, that’s a saving grace, that you can actually see who you’re talking to…maybe we’ve got to get into more technology to get it [so] that people are more linked with people’.

Participants valued the role of commonly available technologies in these contexts; however, they indicated that a general lack of education regarding IT, and the day‐to‐day use of these technologies, remained a major barrier to utilising these tools effectively.

## 4 DISCUSSION

Given the number of weeks spent in COVID‐19 lockdowns in NZ, particularly for people over the age of 70, there was concern regarding the potential for physical deconditioning, isolation and negative psychological impacts.^7^ Whilst not being formally locked down for a longer period, adults over the age of 70 were strongly encouraged to remain at home for several weeks prior to the beginning of the March 2020 lockdown. However, many participants reported increased physical activity and that the negative consequences of lockdown were more impactful on mental well‐being. Levels of both physical activity and conditioning were perceived by participants to be preserved or even increased. This was at least in part due to an increase in walking for exercise, as going for a walk was one way people could leave home during lockdown and was widely encouraged by the NZ Ministry of Health to promote well‐being.^15^, ^16^ Further, the implementation of alternative forms of exercise, including SAYGO exercise equivalents, contributed to the maintenance of physical activity in our cohort.

Psychological distress secondary to social isolation and uncertainty during COVID‐19 lockdowns is a documented phenomenon amongst older persons and the wider population.^7^ Predominantly, SAYGO participants coped well with the mental and social challenges of lockdown, displaying resilience and increasing connectivity with each other. Strong social support networks or being comfortable functioning independently were cited as protective factors. Social isolation was linked to psychological distress, particularly amongst those who lived alone. These findings illustrate the importance of both internal and externally driven coping methods and identifies locus of control^17^ as a key predictor of how individuals respond in the face of adversity. Distress has been previously correlated with perceived relative social isolation, not only with regard to strong, long‐standing social relationships, but also in the context of casual contacts or ‘weak ties’.^18^ This is very relevant in the context of SAYGO, where a range of both strong pre‐existing friendships and more casual social relationships are formed. This is concordant with previous findings, suggesting that SAYGO classes, and the social connections they foster, could enhance the psychosocial well‐being of older persons during lockdowns.^11^

**FIGURE 1:**
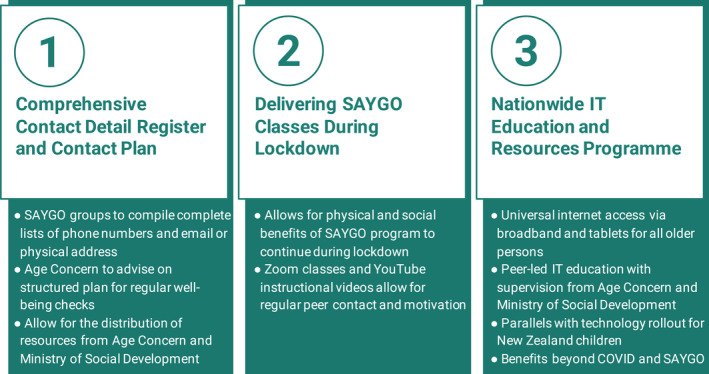
Panel of recommendations to mitigate the impact of future lockdowns. We present three recommendations to address the major challenges identified by our SAYGO study population during potential future lockdowns. (1) Developing a comprehensive register of contact details for SAYGO group members, along with a structured contact plan for future lockdowns, which includes both logistic updates and regular well‐being checks. (2) Providing a number of alternative methods for delivering SAYGO classes, or equivalents, via Zoom, YouTube, or CD. (3) Implementing a national‐scale IT education and resource provision program for older adults, to facilitate increased connectivity for this population both during, and outside of, further lockdown periods

Peer‐leaders from each SAYGO class noted that they were aware of the potential loss of in‐person social contact. To ensure the well‐being of their class members, they attempted to contact them by phone. Participants spoke of the strengthening of relationships via regular contact over lockdown, a sort of ‘coming together in the face of adversity’, a phenomenon, which has also been documented in other emerging COVID‐19‐related research.^8^ For many groups, however, the maintenance of this contact was troublesome, representing one of several difficulties experienced by SAYGO members during the lockdown period. The SAYGO groups experienced a range of challenges to their functioning, including social isolation and difficulty distributing resources. Many participants offered suggestions to overcome these challenges in a future lockdown. Based on these suggestions, we have three recommendations for SAYGO groups and similar community‐based classes to help prepare for future lockdowns (Figure 1).

### 4.1 Development of a comprehensive contact detail register and plan

We recommend that all community‐based, peer‐led groups such as SAYGO develop a complete contact register including phone numbers and email, or physical addresses. This will allow for regular contact as required and ensure that resources can be effectively distributed. A robust contact plan for future lockdowns also needs to be developed, involving regular phone contact for logistics updates and perhaps more importantly, well‐being checks.

### 4.2 Delivering classes during lockdown

Building on the YouTube video created by one of the Age Concern managers, we recommend that Age Concern and SAYGO co‐ordinators improve the accessibility of group members to instructive exercise CDs, so that individuals can continue exercises in lockdown situations.^10^, ^11^, ^19^, ^20^ Further, as the social aspect of SAYGO is a major driver of its success and heavily valued by the participants,^11^ we also recommend the development of resources to facilitate online classes, utilising platforms such as Zoom, to allow members to see and encourage each other in real time. Modifications to the delivery of these classes as appropriate must ensure that such exercises can be safely performed at home.

### 4.3 Nationwide IT education and resources program

We recommend the development and implementation of a national program for IT education and the delivery of IT resources to older people. During lockdown, technology was widely utilised to encourage physical activity and social contact with positive results.^21^ Our findings indicate the need for a nationwide IT education and resources program for older adults. The primary goal would be to deliver universal technology and Internet access, with tablet and broadband provision, facilitating IT education in collaboration with IT education experts and organisations such as the NZ Ministry of Social Development. This would allow for group exercise during lockdown and, importantly, would facilitate communication with friends and family, alongside other social benefits. This proposal parallels the recent NZ government‐funded technology roll‐out in schools, which allows for ubiquitous Internet and computer learning for NZ children. A similar policy in NZ and across Australasia for older adults would have significant benefit for older adult populations.

Implementing these three strategies would provide real benefits in future lockdowns and could be applied in many countries that continue to face significant COVID‐19 restrictions. Furthermore, technological empowerment and increased accessibility would extend beyond lockdowns, to enhance the well‐being of older persons on a broader scale.

### 4.4 Strengths and limitations

Our participant demographics largely reflected that of a typical SAYGO class, where male and Māori participation is often low (M. Dando & C. Tarry, 2021, personal communications). Whilst our participants came from two NZ cities, thus covering potential regional variations in lockdown experience, wider regional coverage would have been desirable. We interviewed a sufficient number of participants to achieve data saturation. As SAYGO is a peer‐led community‐based exercise program, our positive findings relating to maintaining physical activity and function are generalisable to individuals who are motivated to maintaining their physical activity and may not be applicable to older adults in general.^22^

## 5 CONCLUSIONS

The SAYGO fall prevention classes and class members were impacted by the 2020 COVID‐19 lockdowns in NZ. Although physical function was largely maintained in our cohort, psychological distress secondary to social isolation was a prominent concern. Difficulties in maintaining adequate contact between group members, performing exercise classes remotely and optimising the use of technology were major challenges during this time. Accordingly, we have developed three recommendations: (1) develop a comprehensive contact register and contact plan; (2) deliver remote classes over lockdowns using a range of suitable platforms; and (3) implement a ubiquitous technology and IT education program that encompasses older people. These strategies would be invaluable in mitigating the negative impacts of regional or nationwide lockdowns on class members, with implications for the broad and growing population of older adults.

## CONFLICTS OF INTEREST

Professor Debra Waters is the Editor‐in‐Chief of the Australasian Journal on Ageing. No other conflicts of interest declared.

## Supporting information

Table S1

## Data Availability

The data that support the findings of this study are available on request from the corresponding author. The data are not publicly available due to privacy or ethical restrictions.
