# Supplementary material for: Impact of the 2020 New Zealand COVID‐19 lockdown on participants in a community‐based, peer‐led fall prevention program
Source: Australas J Ageing. 2022 Feb 4;41(3):e240–8. doi: 10.1111/ajag.13040 (PMC9111220; doi:10.1111/ajag.13040)
Supplement: Supplementary file 1 — Table S1 [file AJAG-41-e240-s001.docx]

**Table S1.** Pre-prepared guiding questions for interviewers.

| **Role** | **Guiding Questions** |
| --- | --- |
| **Class Member** | - How frequently were you in contact with peer-leaders during the level 4 and level 3 lockdown? - How frequently were you in contact with other class members during the level 4 and level 3 lockdown? - What kind of things were discussed? 1) with peer leaders and 2) with other class members - Did a closer relationship/friendship develop out of this contact with peer-leaders and class members? - Did the lockdown impact on the classes when they resumed, and if so, how? - Did you start the class at the same level of difficulty as before the lockdown? - Have you seen an improvement in yours or other class members’ physical function and overall well-being now that SAYGO classes have resumed? - What types of physical activity were you doing during the level 4 and level 3 lockdowns? - Did you feel that your physical functioning changed during lockdown? - What recommendations would you make for SAYGO classes if the Otago region had a COVID19 recurrence and needed to return to a lockdown situation? |
| **Peer Leader** | - What made you decide to contact class members? - How frequently were you in contact with class members during the level 4 and level 3 lockdown? - What kind of things were discussed? - Were any physical or mental health issues raised by class members? If so, did you need to follow-up with Age Concern? - Did a closer relationship/friendship develop out of this contact with class members? - Did class members also phone or contact you? - Did the lockdown impact on the classes when they resumed, and if so, how? (e.g. were people needing to take more breaks, were they doing exercises at the same level, did you still follow the CD?) - Did you start the class at the same level of difficulty as before the lockdown? - Have you seen an improvement in class members' physical function and overall wellbeing? - What types of physical activity were you doing during the level 4 and level 3 lockdowns? - Did you feel that your physical functioning changed during lockdown? - What recommendations would you make for SAYGO classes if the Otago region had a COVID19 recurrence and needed to return to a lockdown situation? |
| **Age Concern Manager** | - What support did ACO provide to SAYGO peer-leaders during the COVID19 level 4 and level 3 lockdown? - Did SAYGO peer-leaders contact you during the lockdown? If so, for what reason(s)? - What lessons were learned from the SAYGO peer-leaders on how to handle a situation like this in the future? - Were there lessons learned from the SAYGO class members that could be used for future lockdown situations? |
